# How health professionals regulate their learning in massive open online courses^☆^

**DOI:** 10.1016/j.iheduc.2016.07.005

**Published:** 2016-10

**Authors:** Colin Milligan, Allison Littlejohn

**Affiliations:** Caledonian Academy, Glasgow Caledonian University, Glasgow G4 0BA, United Kingdom

**Keywords:** Massive open online courses, Self-regulated learning, Professional learning

## Abstract

Massive Open Online Courses (MOOCs) are typically designed around a self-guided format that assumes learners can regulate their own learning, rather than relying on tutor guidance. However, MOOCs attract a diverse spectrum of learners, who differ in their ability and motivation to manage their own learning. This study addresses the research question ‘*How do professionals self-regulate their learning in a MOOC?’* The study examined the ‘Fundamentals of Clinical Trials’ MOOC offered by edX, and presents narrative descriptions of learning drawn from interviews with 35 course participants. The descriptions provide an insight into the goal-setting, self-efficacy, learning and task strategies, and help-seeking of professionals choosing to study this MOOC. Gaining an insight into how these self-regulatory processes are or are not enacted highlights potential opportunities for pedagogic and technical design of MOOCs.

## Highlights

• Empirical research focusing on individual learner experiences in MOOCs is needed.
• Learners vary in the extent to which they self-regulate their learning in this MOOC.
• Learners' goals and motivation shape their learning behaviour within MOOCs.

## 1 Introduction

The invention of the Internet provided opportunity for radically new models of online learning (Anderson and Dron, 2010, Garrison, 1997). However, online learning provision has tended to mimic conventional teaching in an online setting and models of online learning have largely been adaptations of conventional approaches to teaching, rather than new innovations. For example in Higher Education, campus-based universities tend to use online learning as a complement to face to face instruction, while open universities have largely applied models of distance education that move from the delivery of paper-based materials to online distribution of digital content (Anderson & Dron, 2010). Over the last few years, Massive Open Online Courses (MOOCs) have emerged as a way for millions of learners worldwide to access learning opportunities more flexibly with the advent of thousands of courses, attracting millions of learners (Shah, 2015). While the original proponents of MOOCs envisaged them as a radical departure from conventional, online learning (McAuley, Stewart, Siemens, & Cormier, 2010), the enormous growth of MOOC offerings has been through the emergence of courses that adopt more traditional pedagogical approaches, prioritising scale over pedagogical innovation (Haggard et al., 2013). There are two distinctive features of MOOCs that differentiate them from other forms of online learning: that they offer open access to Higher Education for learners irrespective of their previous qualifications or experience; and that they facilitate learning on a massive scale with thousands, or even tens of thousands, of learners signing up for each course. To enable learning at such scale, and reduce the cost of learning support, MOOCs tend to be designed around a self-guided format that assumes learners are able to regulate their own learning, rather than relying on instructor guidance (Margaryan, Bianco, & Littlejohn, 2015). However, MOOCs attract a diverse spectrum of learners, who vary in their ability to regulate their learning (Halawa et al., 2014, Milligan et al., 2013). The capacity to self-regulate learning is influenced by personal psychological (cognitive and affective) and environmental factors (Zimmerman, 2000a). There is evidence that self-regulated learners adopt effective learning strategies in conventional, online contexts, planning, monitoring, and coordinating their sources of learning (Bernacki, Aguilar, & Byrnes, 2011). MOOCs, however, are qualitatively different from conventional, online courses, particularly in terms of their scale and openness. Gaining insight into self-regulated learning of individual participants in MOOCs is critical in understanding whether and how open, online courses are effective in supporting learning.

This qualitative study examines how learners regulate their learning in a MOOC. The context of study is the Fundamentals of Clinical Trials MOOC offered by edX, a leading provider of open, online courses based in the United States. The study explores the research question: *How do professionals self-regulate their learning in a MOOC?* by collecting and analysing narrative accounts of learning provided by health professionals participating in the MOOC. The paper begins with a review of current research in MOOCs, focusing on studies that address aspects of SRL and further our understanding of MOOC learning. This review is followed by a description of the design and context of this study, and of the instrument used. The results are then presented and discussed. The paper concludes with a discussion of the main findings and their implications, alongside a reflection on the limitations of the study and prospects for future research.

### 1.1 Literature review

The past decades have been marked by changing societal expectations around access to Higher Education. The internet and digital technologies have been viewed as a potential means of opening access to Higher Education to people irrespective of their previous educational experience (Daniel, 2012). However, there is a tension between cost and scale, and universities have sought ways to provide cost-effective access. MOOCs have been promoted as a potential solution to the cost-scale conundrum (Daniel, 2012). MOOC providers, such as edX, Coursera, and FutureLearn, have worked in partnership with universities to provide scalable solutions by designing courses that foreground content presentation, typically lecture video and automated assessment, over opportunities for interaction (Anderson, 2013, Margaryan et al., 2015). This design has led some authors to question the utility of MOOCs as an effective environment for online learning (Rhoads, Berdan, & Toven-Lindsey, 2013). Nevertheless, MOOCs have become a popular choice for individuals seeking learning opportunities, and this has stimulated research effort focused on understanding learning within MOOCs.

While initial MOOC research was often qualitative, quantitative studies have become dominant with the emergence of large scale MOOC platforms that permit the generation and analysis of ‘clickstream’ data (Veletsianos, Collier, & Schneider, 2015). Attempts to interpret clickstream data include mining the data tracking learners' access to MOOC resources and classifying learners according to their patterns of interaction with content (Kizilcec, Piech, & Schneider, 2013) or with other learners in online discussion forums (Gillani & Eynon, 2014). Other studies have focused on MOOC participants' prior education, gender and geographic location (Breslow et al., 2013, Guo and Reinecke, 2014, Kizilcec et al., 2013) to explore the factors underlying poor rates of completion that are typical of MOOCs (Jordan, 2014). But while these quantitative studies of learner activity within MOOC platforms provide us with greater understanding of *what* populations of learners do within MOOCs, our understanding of *why* individual MOOC participants learn as they do, and *how* they actually learn is less developed (Veletsianos et al., 2015, p571). Unlike in traditional HE courses where learner expectations are largely standardised (for example successful completion of a course or degree programme as a marker of success), the diversity of learners in a MOOC results in a range of motivations for participation (Kizilcec et al., 2013) and potentially leads to different levels of engagement (Breslow et al., 2013) which may not be focused on completion. In a MOOC, where certification may be absent, or of little value (Kizilcec et al., 2013), learners are required to be more intrinsically motivated, recognising their own goals and indicators of success. Breslow et al. (2013) argue that it is important to understand the influence of learner motivation on learning in MOOCs. Similarly, Gašević, Kovanović, Joksimović and Siemens (2014, p168) call for studies that improve our understanding of ‘motivation, metacognitive skills, learning strategies and attitudes’ in MOOCs arguing that because levels of tutor support are lower than in traditional (formal) online courses, there is a need for greater emphasis on the individual learner's capacity to self-regulate their learning. Self-regulation is the ‘self-generated thoughts, feelings and actions that are planned and cyclically adapted to the attainment of personal goals’ (Zimmerman, 2000a, p14). Zimmerman identified a number of components (sub-processes) of self-regulation including goal-setting, self-efficacy, learning and task strategies, and help-seeking. Although originally conceptualised in formal (classroom) settings, SRL and its sub-processes have subsequently been studied extensively in online contexts (see Bernacki et al., 2011 for a comprehensive review) and SRL is increasingly being used to investigate learning in MOOCs. Research that explores these aspects of SRL in MOOCs is described below.

Zimmerman (2000a) highlights goal-setting as a central component of SRL. By setting goals, the learner is able to monitor progress towards those goals, adjusting their learning as necessary. Different types of goals are recognised, ranging from specific, learning focused goals driven by intrinsic motives to extrinsically motivated performance goals (Pintrich & de Groot, 1990). Setting goals and monitoring them is motivational as it provides evidence of progress to the learner. Haug, Wodzicki, Cress, and Moskaliuk (2014) explored the utility of badges in a MOOC focused on emerging educational technologies. The authors used self-report questionnaires and log files to explore patterns of participation, and found that learners who had set a goal to complete the course were more likely to sustain their participation (determined by measuring access to course content and active engagement with others about the course) than those who did not set a goal. Completion of the course provided an extrinsic motivation for these learners (Ryan & Deci, 2000). However, as highlighted above, MOOC learners may not be motivated by completion, so it is important to understand different types of motivation for MOOC study. Zheng, Rosson, Shih, and Carroll (2015) conducted interviews with learners who had undertaken a variety of MOOCs and identified four categories of MOOC learner motivation: fulfilling current needs, preparing for the future, satisfying curiosity, and connecting with people. Their findings suggest that completion is just one outcome of MOOC participation, with key motivations to study being intrinsic in nature, related primarily to personal improvement. In a larger, survey based study, exploring motivations of MOOC learners based in the United Kingdom, Spain and Syria, seven different types of motivation were identified (White, Davis, Dickens, Leon, & Sanchez Vera, 2015), mirroring the categories identified by the Zheng et al. (2015) study, and in addition identifying categories of motivation reflecting other extrinsic factors: the free and open nature of MOOCs, their convenience, and the prestige of courses run by high quality institutions. These studies identify the types of goals learners may be setting, but do not tell us about how different types of goals influence learning in MOOCs.

Self-efficacy, the personal belief about having the means to perform effectively in a given situation (Bandura, 1986), represents another component of self-regulation. An individual's self-efficacy influences how they respond to setbacks in their learning, with highly self-efficacious individuals redoubling their efforts in an attempt to meet their goals when faced with a challenge, while those lacking self-efficacy may give up or become negative (Zimmerman, 2000a). In a study of learners registered for a MOOC on economics, Poellhuber, Roy, Bouchoucha, and Anderson (2014) explored the relation between self-efficacy and persistence using clickstream data and scales for self-efficacy and self-regulation. Their study found a positive link between self-efficacy and persistence, though the main predictor they identified was initial engagement. Wang and Baker (2015) studied participants on a Coursera MOOC on big data in education to explore the link between motivation, self-efficacy, and completion. The study found that participants who self-reported higher levels of self-efficacy at the outset of the course were more likely to persist to the end, echoing findings from online learning research (Wang & Newlin, 2002). Our own parallel study of participants in a MOOC on Data Science (Littlejohn et al., 2016a, Hood et al., 2015) linked a range of factors: previous experience of MOOC learning, familiarity with content, and current role to learner self-efficacy.

Learners draw on a range of cognitive and metacognitive strategies (learning and task strategies) to support their learning, including taking notes, revising, supplementing core learning materials, exercising time management and undertaking on-going planning and monitoring. Highly self-regulated learners draw on a wider range of strategies and recognise the applicability of different strategies to different situations (Zimmerman, 2000a). They are also able to effectively monitor their learning, changing strategies when they become ineffective. Veletsianos et al. (2015) explored the learning strategies of a small group of learners who had completed at least one MOOC, focusing on note-taking and content consumption. Their interviews uncovered a variety of note-taking strategies that facilitated these individuals' engagement with the course content. The range of note-taking strategies utilised illustrated how different approaches such as taking digital notes, using a dedicated notebook, or annotating printed slides, complemented different patterns of participation and engagement. Other learning and task strategies are also important. For example, in a survey-based study exploring the causes of high drop-out rates in MOOCs, Nawrot and Doucet (2014), identified time management as the primary reason for MOOC drop-out, being cited by more than half of their survey respondents, though their study did not collect detailed descriptions of how time management skills contribute to effective learning in MOOCs.

Help-seeking: recognising the limits of one's own knowledge and understanding the role that others can play in one's learning is another key attribute of self-regulated learners. Studies by Cho and others have demonstrated that learner interaction such as seeking help is important for high quality, online learning (Cho and Kim, 2013, Cho and Jonassen, 2009). The importance of learner interaction was also highlighted by Abrami, Bernard, Bures, Borokhovski, and Tamim (2011) in their meta-analysis of a range of studies of distance education and online learning. That study concluded that online learning designs should incorporate learner interaction, but that such an approach is dependent upon learners having the capacity to self-regulate their learning. MOOC researchers have explored the impact of social interaction on MOOC learning. In a small scale case study, Chen and Chen (2015) looked at how participation in a local face-to-face study group improved motivation, broadened perspectives, and led to shared learning strategies among MOOC learners. Interaction with peers can also be effective online. Gillani and Eynon (2014) established a link between forum participation and MOOC completion by analysing patterns of interaction of highly performing students in a MOOC focused on business strategy. Learning can occur with other students in the same cohort, or with others in existing networks. Veletsianos et al. (2015) describe how learners in their study who took digital notes shared them with their peers through social networks. The study, which focused on interactions which took place outside course platforms, found that learners consistently described these learning focused social interactions as meaningful, though the authors concede that their analysis was unable to provide an insight into how these interactions affect learning.

In addition to these studies that use concepts of SRL to explore individual learning in MOOCs, a complementary strand of MOOC research has used SRL to critique and inform MOOC design. Bartolomé and Steffens (2015) used SRL as a lens to critically evaluate the utility of MOOCs as a learning environment. Their theoretical study applied criteria originally developed to evaluate online learning platforms (Steffens, 2006) and concluded that MOOC platforms such as those offered by Coursera and edX could be categorised as a ‘content system without tutor’ supporting cognitive and motivational components of SRL, but providing little support for emotional and social components of SRL. This analysis highlights the inherent shortcomings of MOOC platforms, and signals the type of skills that learners need to possess and use to learn effectively in these courses. Gutiérrez-Rojas, Alario-Hoyos, Sanagustín, Leony, and Kloos (2014) argue that the lack of interaction opportunities offered by these MOOC platforms disadvantage learners who have poor study skills and may contribute to early drop-out seen in MOOCs. To address this inherent shortcoming of MOOC platforms, Gutiérrez-Rojas et al. (2014) designed a mobile application that supported novice learners as they studied on a MOOC, scaffolding their interaction with content and replacing some of the functions of a tutor. The design of the application is mature, but its effectiveness has yet to be evaluated (Alario-Hoyos, Estévez-Ayres, Sanagustín, Leony, & Kloos, 2015).

## 2 Study rationale

The studies described above suggest that self-regulation is important for effective learning, and that learners differ in the extent to which they self-regulate their learning. However, these studies give little insight into the actions and behaviours learners adopt to learn in open, online, non-formal contexts. This study builds on earlier work examining the self-regulated learning undertaken by professionals in a variety of contexts (Littlejohn et al., 2016a, Hood et al., 2015, Milligan et al., 2013, Milligan and Littlejohn, 2014b) to investigate how professionals self-regulate their learning in the context of a MOOC. The study design utilises a qualitative SRL instrument to reveal narrative accounts of learning from participants in the MOOC and through them to identify patterns of self-regulation.

### 2.1 Context and method

The *Fundamentals of Clinical Trials* MOOC, (https://www.edX.org/course/harvard-university/hsph-hms214x/fundamentals-clinical-trials/941) provided an introduction to the research designs, statistical approaches, and ethical considerations of clinical trials. The 12 week course was aimed at health professionals and those studying for a health professional role and attracted 22,000 registrants from 168 countries. Participants for the study were drawn from a larger cohort of learners who responded to a message posted to the course website in week four (November 2013) inviting them to complete a survey instrument designed to provide a measure of their self-regulation. The makeup of the study cohort (n = 350) was representative of the overall demographic profile of the course cohort (source: HarvardX insights: http://harvardx.harvard.edu/harvardx-insights) in terms of gender, age, education background and geographical distribution.

Participants who completed the survey instrument, and who identified as healthcare professionals (n = 126), were invited to take part in a semi-structured interview designed to explore their self-regulated learning in the MOOC using a script developed iteratively over a number of studies (Milligan et al., 2013, Littlejohn et al., 2016b, Milligan and Littlejohn, 2014a, Littlejohn et al., 2016a). Relevant questions are included in the Section 3 below, with the full interview script available online (Milligan & Littlejohn, 2014b) . Thirty-five Skype interviews were conducted during November and December 2013. The interview transcripts were analysed to probe how participants' self-regulate their learning in relation to each of the sub-processes described by Zimmerman (2000a). Each of the 35 transcripts were coded independently by two researchers, and codes assigned corresponding to these SRL sub-processes as well as other coding structures reported separately (Milligan & Littlejohn, 2014a). Discrepancies in the coding between the two researchers were minor and were resolved prior to the commencement of a second round of analysis. The transcripts were then re-analysed by two researchers (independently, then jointly, to reduce the risk of bias) to identify emergent patterns of self-regulated learning behaviour.

## 3 Results and discussion

This section describes the analysis of data from the interviews, arranged thematically by SRL sub-process. The transcript analysis uncovered detailed accounts describing participants' goal-setting, self-efficacy, learning and task strategies, and help-seeking. These accounts are presented in turn below with a summary and initial synthesis at the end of each sub-section. The interview questions did not elicit detailed descriptions of the other SRL sub-processes (task interest and value, interest enhancement, self-satisfaction, and self-evaluation) and these sub-processes are not discussed further. Table 1 lists the study participants, their gender, role, and geographic location.

### 3.1 Goal setting

Descriptions of goal-setting and identification of diverse types of learning goals were elicited through questions including: *Can you summarise your main aim in this MOOC?* and *Did you set specific goals at the outset of this MOOC?* Most participants (28/35) described setting goals. 21 participants described goals focused on what they aimed to learn from the course, while 19 participants described performance goals focused on their completion of the course or attainment of the course certificate. There was some overlap between these two groups with 12 participants describing both learning and performance focused goals.

#### 3.1.1 Learning goals

Goals focused on learning primarily articulated how the course content related to, or enhanced career prospects or job requirements. A Nurse Teacher (participant 358) described how the course complemented his existing knowledge and skills in his current role:

“I know the material, but I need and I am looking for different explanations of the syllabus. So these open courses are giving me helpful information of how to resolve and how to explain the same issues in another way.”

Similarly, when asked about her goals, a Clinical Research Consultant (participant 373) clearly indicated how she expected the course to supplement her existing knowledge:

‘It's learning, getting to know more about some things that I already know, but I wanted to go into more depth, to get more *information* because there are some areas that I am not good at, like biostatistics, even study the design because I was not doing that a lot.’

Most goals were articulated at this broad level, with only a few participants reporting goals focused on specific aspects of the course. A Physician (participant 295) described a narrow learning-focused goal: ‘*My goal was to be very confident of (the) fundamentals of probability in clinical trials*.’ This learner had already taken three statistics MOOCs and was focused on addressing a particular gap in her knowledge.

#### 3.1.2 Extrinsic goals

Other learners described goals that focused solely on extrinsic criteria – completion of the course or attainment of the certificate – making no detailed reference to what they expected to learn. A Neurologist (participant 249) described how his goals were ‘*… to watch the videos and access the learning material and then to gain the certificate*.’ These non-specific goals could apply to studying on most MOOCs and do not indicate the same level of engagement as focused learning goals. The Fundamentals of Clinical Trials course originated at Harvard Medical School, and several respondents, such as this R&D Innovation Projects Coordinator (participant 152), articulated goals focused on certification of their learning: ‘*The goal is to have an in depth knowledge of this area from a very prestigious university like Harvard and having it certified with a certificate, it will be great for me and my job afterwards*.’ This ‘Harvard brand’ was attractive to many.

#### 3.1.3 Performance goals

Twelve respondents articulated both learning-focused and performance-focused goals. A Clinical Trials Project Manager (participant 255) listed two goals, the second clearly articulating how it would benefit her future work:

‘*I was expecting to be able to complete the course first of all and second to have an overview from A–Z. I was not expecting to learn a lot of things in depth, which is normal.’* adding *‘But my two objectives were an A-Z learning/understanding and to understand better my day to day work, my day to day practice at the organisation of clinical trials where I work*.’

Similarly, 366 (Lecturer, ranked 5 for SRL, 7 = for goal-setting) described her goals and her ambition to expand her learning:

“The first goal is to pass and get the certificate of achievement… second goal is to participate in the discussion forums as much as I can, try to benefit from the teaching assistants and professor and understanding new things that I didn't know before”.

The personal nature of this goal was unusual, with few participants focusing explicitly on their intrinsic motivation. One other example is from a Paediatric Pharmacist (participant 334), who, while acknowledging the attraction of certification, indicated that her primary motivation was simply to ‘learn from the best’:

*‘I would like to have finished the class, to get the certificate, but it wasn't really for that. I think it's more personal, like a personal goal, like I just wanted to learn from the best. So it's great that you have a certificate, but I'm not about the piece of paper, I'm about the learning opportunity.’*

#### 3.1.4 Non-explicit goals

Finally, a small number of participants (7/35) appeared not to have set goals, though in response to the question, most of this group articulated goals based on completion or certification. For example, the Physician (participant 213) responded to the question of whether she had set any goals with ‘*I didn't. I tried to get through the course.*’

#### 3.1.5 Summary

In summary, most participants articulated goals, but these goals varied greatly, with some respondents focused on extrinsic outcomes, such as course participation, completion and certification, while others articulated more specific goals related to course content, or the intrinsic benefits of their study to their career, current role or personal satisfaction. Goals of any type can be motivating, though intrinsic goal orientation is more strongly associated with academic achievement in online contexts (Cho & Shen, 2013). The range of goals identified in this study matches the motivation types identified by White et al. (2015). Only a very small number of participants set goals relating to mastery of specific concepts of expertise development. Instead the learning goals articulated more general descriptions making reference to the overall course topic. In this course, learning objectives provide a clear structure to the course for all participants, and across the groups there was a clear awareness of the course content. The objectives are intended to guide the learner's participation in the MOOC, by signalling what the learner should learn. However these objectives might also encourage learners to adopt a passive approach to their learning, viewing and reading the learning material without engaging in learning activities that fulfil their original learning needs.

### 3.2 Self-efficacy

Interview transcripts were analysed for indicators of self-efficacy. Questions designed to probe self-efficacy included: *Do you feel able to manage your learning in this MOOC?* and *Do you feel able to integrate your learning on this course with your professional practice?*

#### 3.2.1 Signs of confidence

The majority of learners interviewed (28/35) provided accounts that demonstrated good self-efficacy. These participants typically provided clear and detailed descriptions of their learning. For example, when asked about his experience of this course, a Medical Laboratory Scientist (participant 394) reported no problems with his learning: ‘*Yes I think I have been quite comfortable doing it. I work full time, I study part time in my own time, but yeah I have really had no problems*.’ He then went on to indicate recognition of his individual responsibility as a learner:

‘I knew it was going to be a course which would be taking quite a bit of my time … there was a lot of material which I had to cover, so I knew I had to commit myself … and actually find time for the course.’

This inherent understanding of how the MOOC fitted into their on-going learning was also evidenced during the interview of an R&D Innovation Projects Coordinator (participant 152) who described how the course helped her expand her existing knowledge: ‘*I′m very familiar with the subject, I already have a good background, I have all the resources and knowledge about this issue … that's why it's not hard for me to grasp what they say in the lectures*.’ She then affirmed her confidence in her own ability and persistence: *‘Actually it's related to (your) character and personality and commitment. I'm this kind of person if I have a commitment to have a certificate then I will have a certificate*.’

#### 3.2.2 Lack of confidence

Not all participants were as assured of their ability to learn and succeed in the course. A minority of those interviewed (7/35) provided descriptions of their learning that indicated lower levels of self-efficacy in this context. One Physician (participant, 213) reported having difficulty with learning: ‘*The course material is quite a lot. … I hoped I can get the certificate, but I found it quite difficult for me.*’ Although a health professional, this participant was not currently involved in clinical research and would have been unfamiliar with much of the content of the course, unlike some participants already working in the field who may have a basic understanding and were seeking to formalise their learning rather than learn something entirely new. For other participants, lack of self-efficacy may have been due to a lack of familiarity with the MOOC format. A Clinical Pharmacy Lecturer (participant, 316), also appeared to doubt his ability to learn, but in this case, it was his lack of experience that was important: ‘*sometimes during the course I found myself lost, this is due to the background may be that I was deficient*.’ He indicated a need for assistance: ‘*I always start searching on an internet engine, but it needs some sort of assistance* ….’ suggesting that he would have preferred to have received more guidance in the course.

#### 3.2.3 Summary

Most of the accounts indicated high levels of self-efficacy as may be expected given the background of the participants in this study. However it is clear that some participants were not as confident of their ability to succeed in the MOOC due to their lack of prior experience of MOOC learning or lack of familiarity with the content. These findings reflect those of a companion study (Littlejohn et al., 2016a) which also found that self-efficacy was impacted by previous familiarity with learning content or platform. Self-efficacy is highly context dependent and linked to task familiarity (Zimmerman, 2000b) and experienced MOOC-takers often talked in their interviews about how they had settled on an approach to MOOC learning. Learners without prior MOOC experience would benefit from additional support in the form of tutor guidance, additional resources or orientation to the course environment.

### 3.3 Learning and task strategies

Interview transcripts were analysed to look for the range of learning and task strategies utilised by study participants. Two aspects were probed in particular: whether and how an individual had taken notes (*Questions: Has your learning involved the creation of anything?* and *Did you make any notes for yourself?*), and how active their approach to learning on the course had been.

#### 3.3.1 Active production and passive observation

More than half (20/35) of those interviewed took notes to support their learning in the MOOC, with accounts describing how they contributed to learn in different ways. For the most part, notes were taken as a means of summarising the video lectures, perhaps deploying strategies learned at University. As one Pharmacist (participant 256) reported: ‘*I behave as if I am in a lecture theatre when I'm watching these videos. So I would take the sort of notes that I would have taken at university or any other lecture theatre*.’ These lectures were delivered in English, and for non-native speakers, notes represented an effective way of reinforcing their understanding. A Psychiatrist (participant 371) described how: ‘*I write notes because it is hard for me to understand the videos because they are in English …usually I write down in Spanish*.’ While there were differences in how notes were taken, with some preferring paper and others preferring digital notes, the descriptions provided almost always related to text based notes, with only two reports of non-text based notes. For example, a Lecturer (participant 366) who was using the course to improve the support she could give her students described how she made tables and charts and remarked: ‘*I try to transfer the information to easier forms.*’

Taking notes may be part of a strategic approach to learning. For example, a Nurse Teacher (participant 358) described how he recognised signals in the learning materials, for instance when the lecturer presented highly structured information: ‘*I′m taking notes because the exercise will ask me about these points*.’ He then expressed the value he perceived in note-taking: ‘*Learning is not just watching videos or attending classes, learning is better when the student is pushed to take notes to read and then answer some exercise which will involve the readings and the notes.*’

A minority (15/35) of those interviewed did not take notes. For some, such as this Physician (participant 295), note-taking was not a learning and task strategy she would routinely use:

‘I do download the study material which is provided, but while I watch the video I do not have a habit of making notes and I am a person who is organised in a mess. So even if I make a note I don't recollect and read those notes.’

Similarly, a Clinical Pharmacy Lecturer, (participant 316) recognised the value of note-taking as an aid to learning, yet did not write notes: ‘*Notes, no I didn't make notes … my professor always tells me that you have to take notes and reply and comment and I think this is one of my disadvantages regarding reading and I know it's a deficiency*.’

Sometimes, not taking notes was an active decision. As the course was wholly online, some participants opted simply to collect all the resources (videos, weekly readings) in one place on their computer and save these for future reference. A teacher (participant 324) remarked: ‘*I do it on the computer … but not taking notes of anything. Sometimes I go back to the video, sometimes I print the articles they recommended, so I read these, but I'm not taking notes at all*.’

#### 3.3.2 Responsive adaptation and adaptive learning

To understand learning and task strategies in a broader context, learners' active engagement and how they managed their time in the course was also examined. Analysis focused on how learners matched their effort to the demands of the course and the extent to which they had adapted their learning during the course (Questions: *How did you manage your time? Have you modified your learning approach during the course?*). The course followed a regular structure and learners typically set aside time to watch videos and explore the recommended texts, often fitting in their study around other professional and personal commitments. A neurologist (participant 249) described his study pattern:

‘I do the course at the end of my activities, between 9 and 10 o'clock at night. … I take an hour probably a day to go through the materials and when I have a doubt I read first and then go back to the material.’

Just over half of those interviewed (20/35) appeared not to have changed their approach to the course. For some, this was because they knew what to expect from MOOC study (having studied other MOOCs previously) or due to previous familiarity with course content. For example, a Clinical Research Associate (participant 47) described how she felt ‘*the course is quite easy*’, reporting that she was able to follow the course with a minimum of effort. A minority of this group (6/20) provided descriptions of their learning that indicated that they had faced challenges, yet did not adapt their approach. For three learners, time had been a factor. For example a Consultant (participant 226) recognised the importance of extra reading, but had not earmarked time to read: ‘*I have access to the text book, I should have tried to go through it and learn from it, but I'm not able to give time for that*.’ A physiotherapist (participant 28) reported similar actions: ‘*Yes they provided us with the name of a textbook … I downloaded this book, but I never have time to have a look at it*.’ A Physician in this group (participant 213) repeatedly indicated she found the course challenging. When asked about her intention to complete the quizzes she responded: ‘*I tried to, but some questions I cannot get the correct answers*.’ or when asked about the course reading ‘*In my previous learning I (was not) expected to think about the articles. In this course we have to think more after reading research articles*.’ Despite recognising problems with their learning, these learners did not appear to have changed her approach.

The remainder (15/35) of those interviewed had changed their learning approach during the course. For some, it was clear that MOOC learning was a new experience for them, demanding greater effort than anticipated. A Clinical Trials Project Manager (participant 255) described this in detail:

For me it's new to learn something by watching a video. Sometimes I have to be more critical … it's not A + B = C, I have to think differently and even if I take good notes, I don't find the answer … and I have to go back to the transcript and to the chapter and read again what the professor said and then during your reflection [you] find the solution, find the right answer.

For others, it was not lack of familiarity with the format that had motivated their change, but instead, a recognition of a limitation of their own inherent learning behaviour. For example, a Clinical Research Consultant (participant 373) described how: ‘*I sometimes suffer from procrastination, so I have to make myself do it in a certain time … I made a plan, like an action plan, for each module*’*.*

Finally in this group there were some learners who had adjusted their approach not to address a learning challenge, but rather to match the benefit they felt they were gaining from the course. Two learners described how they had found the forums helpful, and had increased the time they spent reading posts in response. A Nurse Teacher (participant 358) focused his effort on particular aspects of the course that were of interest. He described his strategic approach as follows:

Well every unit I review what's going on. So if I'm very interested in one unit I [devote] more time, so then I read more papers, I read more material, more references, websites and then even I can watch the videos more times. But if I'm not really interested, less motivated, I'm just watching the video, answer the exercise and then go on to the next*.*

#### 3.3.3 Summary

In summary, there was evidence of some participants taking control of their learning, actively modifying their approach and managing their time to match their effort to the benefit they perceived, and to increase the effectiveness of their learning. It also appears that some participants lacked the skills or motivation to monitor effort or manage their time effectively. Nawrot and Doucet (2014) argue that MOOC designs should support effective time management strategies including the provision of example study plans, assigning time estimates to all activities, and providing tools to support learners to schedule and plan their learning. The provision of a scheduling tool in particular could support learners who are less skilled in time management to develop these skills. The accounts of note-taking indicate that this learning strategy was primarily used as a means of summarising video lectures, applying learning skills developed in formal education. Summary notes were used particularly by learners whose first language was not English. Only a few examples of more sophisticated note-taking approaches were reported and although some participants recorded their notes digitally, this did not seem to facilitate sharing in this cohort, unlike the MOOC learners studied by Veletsianos et al. (2015).

### 3.4 Help-seeking

The edX MOOC platform incorporates a discussion forum which acted as a space for a case analysis exercise (those wishing to earn the course certificate were required to contribute), as well as a locus for informal (and optional) course related discussion. Transcripts were analysed to identify indicators of positive and negative attitudes to learning with others as well as accounts of help-seeking and engagement with others during the course. Participants were questioned about interactions both within and outside the course (Q: *Did you interact with other course participants during this MOOC?* and *Have you interacted with others in your external network about the course?*). However, there was little evidence of learners interacting with people within networks outside the MOOC (Milligan & Littlejohn, 2014a). The analysis presented here is, therefore, focused on discussion forum activity. All participants had an on-going opportunity to interact through the course discussion forums, either through actively posting questions or providing answers, or by choosing instead to observe others. While all participants interviewed had looked at the forums, only around half had actively participated in the forum (17/35), with most of this group (12/17) recognising the clear benefit it provided, as illustrated by this quote from a Physician, (participant 318) describing its overall value: ‘*a lot of people from different backgrounds will be coming to the course, which is definitely an advantage over an offline course.*’

#### 3.4.1 Helpful learner dialogue

For those with the skills to interact with others, the forum could be a valuable source of learning, as reported by another Physician (participant 295). When asked whether she interacted with other course participants, she responded:

*‘I do it every day. My experience with the MOOC so far is equal learning, if not more, happens in the discussion forum. It is a great place and I make it a point that I visit the discussion board every single day, read through most of the posts and try and participate/share my views as well. It's an amazing place.’*

This benefit of learning from others may be unanticipated: a Medical Epidemiologist (participant 22) described how she had found the forum more useful than she had expected: ‘*at the beginning I was not planning to participate in the forum and as the course went on I am learning more. I mean I read more in the forum and I try to participate*.’

Whatever their intentions, these experienced practitioners were drawn into discussions as they saw that their own experience would be of value to others. A Physician (participant 360) described how she was able to bring her own professional perspective into the discussion, illustrating the potential of this multidisciplinary course:

*‘I found myself commenting on a couple [of posts] just because I knew the answer to their question and a lot of them would talk about just the practice of medicine in general, you know they make comments about how this ethically related to the clinical studies and stuff like that and so I wanted to give it a perspective from my educational background which is being a doctor, I know what it's like.’*

For some, including this Lecturer (participant 366), the forum was central to their study on the course: ‘*Second goal is to participate in the discussion forums as much as I can, try to benefit from the teaching assistants and professor and understanding new things that I didn't know before.*’ The same Lecturer described how she routinely interacted with peers: ‘*actually today I was thinking to share some thoughts about or some conclusion and collecting some ideas … the first thought was to share it with the MOOC course*.’ Similarly, a Pharmacist (participant 280) described how she enjoyed discussing ideas with other professionals in depth, using the forum space to:

‘give arguments and discuss what you think, what are your experiences. That's a nice thing because this course is a little bit specific… you have [specialists] who know what they are talking about, they are not…I don't know how to say it, civilians that don't understand professional words and that's what I like, something a little bit more serious.’

Interactions in the discussion forum can provide a relational dimension to learning. A Data Manager (participant 284) highlighted how sharing ideas on the discussion board had provided a mechanism for expanding his professional network:

‘ … because we are looking at, even after the end of the course, we'll still keep in touch and create a network for the health workers, those working in health because the course has so many professionals, we have surgeons, we have nurses, we have doctors, we have public health practitioners. So we are looking at creating a network at the end of the course’

#### 3.4.2 Unhelpful learner dialogue

A small number (5/17) of learners who had actively participated in the forums were less positive about their experiences. A Clinical Pharmacy Lecturer (participant 316) appeared to lack self-efficacy, describing his experience negatively as follows: ‘*… you reply to someone who exhibits her ideas regarding maybe a certain question or certain discussion, but no response … I don't know, maybe they are busy, your participation is maybe not convincing to them*.’ A Surgeon (participant 72), was even more negative, eventually giving up on the forum:

‘No one was helpful. Most of them didn't even understand what I meant at all, … I have tried 2 or 3 times to try and explain my problem and they couldn't understand me at all, I gave up and I really honestly don't have the time to spend so much time on the discussion board.’

For the third member of this group, a Clinical Trials Administrator (participant 275) engaging with the forums was not worth the effort: ‘*it's really difficult to find anything specific in there, it seems a bit unorganised and lots of things are repeated. So sometimes you find a really good comment from someone, but it seems to be more a matter of luck*.’ When asked whether she had anticipated using the forum more, she responded: ‘*Yeah I think I did. Also to get the feeling that there were other students as well, but yeah I couldn't really find a useful way to dig into it*.

#### 3.4.3 No learner dialogue

Slightly more than half of the group interviewed (18/35) limited their activity to reading posts by others. In forums where there are large numbers of users, an individual may find the help they need by browsing existing content, rather than actively requesting assistance. The decision not to engage was an active one, with learners finding a level of engagement that suited them. Lack of time was a key factor in choosing how to engage with the forums, and for many, this optional activity was sacrificed in favour of core course activities. A Physician (participant 318) described how time had limited her activity: ‘*If I had more time I would have interacted more with the forums, but time was a little problem, so that is why I couldn't interact much more*.’ All of the participants interviewed were working as health professionals and few had been permitted to set aside time to study by their employers. For others, not posting reflected the preference of individual learners. For example, a Psychiatrist (participant 371), appeared to have limited her interaction with others because of the platform design rather than any negative views of learning with others: ‘*I haven't discussed with anybody because it isn't a good format to discuss*.’ She went on to suggest that real time discussions, with teachers, would have been more useful for her learning, a type of interaction that is not possible in a MOOC of this scale.

When asked whether she had interacted with others, a Clinical Research Consultant (participant 373) replied ‘*I am weak in that area because I don't like chatting online and sending messages and participating in discussion boards*.’ indicating that her problem was not caused by the edX MOOC platform. Here, the underlying reason could be cultural as this Serbian participant, who had previous experience of online study (to Masters Level) stated:

‘where I live, … there is a totally different attitude to learning. When you go to school you are served a certain amount of information and you are supposed to memorise them. You are not supposed to learn to think and to participate in discussion.’

For some participants, there was a clear preference to learn alone. A Medical Laboratory Scientist (participant 394) described how: ‘*I prefer to work alone … the only time I have visited the discussion boards would be when I have really run out of ideas and to get clues of what others may have.* A Paediatric Pharmacist (participant 334) expressed a similar negative view:

‘you know if you learn from other people who don't know what they're talking about you could teach yourself the wrong thing. So my focus is [on the researchers], I read them [discussion posts] but I take them with a grain of salt, I'm like “I don't know if this person knows what they're talking about”. So I just keep the information that the researchers are telling me and then I'll use that for my own knowledge.’

Elaborating on her attitude to help-seeking, she remarked: ‘*I′ve never really been a study group person, I′ve always been a study group leader … I′ve always kind of worked with them to help them*.’

#### 3.4.4 Summary

In summary, around half of those interviewed participated actively in the forum, and for most this had been a positive experience. These learners saw the potential value of learning from one's peers, and how this could broaden their learning. Other participants recognised the role they could play in passing on their professional experience to others and the role of the discussion forum in growing their learning network. A small number of participants had negative experience of the forum, expressing frustration at the quality of discourse, or the utility of the platform. Half the participants did not actively engage in the forums but instead utilised the forum simply as a source of information (often for reasons relating to time), with a few participants harbouring explicit reservations about learning from their peers. Unlike Veletsianos et al. (2015) this study found little evidence of learners using social networks outside the course environment to support their learning. Indeed in this MOOC, there was little evidence of learners talking about their learning outside the course even within their face to face professional networks (Milligan & Littlejohn, 2014a). There may be two explanations for this. First, Veletsianos et al. (2015) recruited their participants through social networks and may have self-selected a sample comprised of enthusiastic users of social networks. Second, the highly structured nature of the Fundamentals of Clinical Trials MOOC may have encouraged participants to perceive it as a self-contained course.

## 4 Conclusions

This study examined narrative descriptions of learning from MOOC participants, illustrating the range of self-regulation of learning that occurs during MOOC study. There were clear differences in the types of goals set, and help seeking behaviour, with less distinct differences in the learning strategies adopted and levels of self-efficacy reported. The narrative descriptions collected provide an insight into how learning occurs in MOOCS and how learners who self-regulate their learning to different extents might be supported within MOOC platforms.

The different sub-processes of self-regulated learning are of course highly inter-connected, with evidence from quantitative studies using self-report instruments to measure these sub-processes indicating that they are strongly correlated (Littlejohn et al., 2016b, Milligan et al., 2015) in different populations. Bringing together the four sub-process described here, some clear patterns of learning emerge that illustrate their inter-relationship. On the one hand, there are highly self-regulating learners who have a clear understanding of what they want to learn and how it will impact their career, job or personal development. These individuals assume control of their learning, monitoring their progress and adjusting their effort to maximise the benefit they gain from their studies. These learners go beyond the core tasks of the course, searching for additional resources and engaging with others in the forums to develop their ideas and grow their learning network. They are also strategic in their approach and may miss out parts of the course that are of less interest – finishing the course is not necessarily a measure of success for them. At the other end of the spectrum, there are learners in the MOOC that do not seem to be self-regulating their learning to any significant degree. These learners focus on completion and certification as their measure of success, and who appear not to have considered the personal benefit that participation will bring them. These learners are content to closely follow the course structure of video lectures, readings and quizzes, devoting the same amount of time each week, but can become derailed if they begin to find the material more challenging as they are unable or not prepared to change their approach.

MOOCs are positioned as accessible to all. Support should be available for those who lack confidence to interact, or articulate their own expectations of the course, or who do not have strategies to learn effectively in MOOC platforms. For those who are unable or choose not to self-regulate their learning, MOOC platforms could incorporate tools to scaffold learning, and encouraging the development of skills such as time management, goal setting, reflection, and help-seeking, elevating these platforms beyond the ‘content system without tutor’ category described by Bartolomé and Steffens (2015). Tools such as the MyLearningMentor application described by Gutiérrez-Rojas et al. (2014) could scaffold the learning of participants exhibiting low self-efficacy due to lack of familiarity with the content or environment. Extending the self-set badge system described by Haug et al. (2014) to encourage learners to articulate what they want from the course would increase engagement. Such a system could be of particular benefit to learners who would not otherwise have set goals. For those who are highly self-regulated, MOOC environments should seek to be flexible, allowing these learners to take assume greater control of their learning experience: to choose alternate routes through content that suit their specific goals and motivations, to integrate learning content with their existing knowledge, and to share their learning with peers within and beyond the course boundaries. The eLDa platform, developed at the University of Warwick (Onah & Sinclair, 2015) incorporates some of these features, while also providing a more highly structured environment to suit learners who require more support.

Improving our understanding of the range and underlying basis of learning in MOOCs will enable designers to design more supportive learning environments and effective learning tasks (Littlejohn & Milligan, 2015). Despite the variations in learning observed in this study, all learners were persisting with the course and almost all confident of completing it. This may be due in part to the design of this MOOC that focused on content delivery. The course objectives provided a clear set of goals to follow that would ensure completion, the course content provided all the information necessary to complete the course tasks (multiple choice assessments) and aside from two compulsory discussion forum tasks, there was no requirement for participants to interact with their peers. The platform and course design may be effective at content delivery, but a question remains over its utility as an environment for learning.

### 4.1 Limitations and further research

There are some inherent weaknesses within the design of the study. First, only a single MOOC was studied. Without repeating the study in other MOOC contexts, there is no way of knowing if the range of learning patterns and strategies reported here would be observed in a different MOOC context, particularly one where demands on learners to manage their own learning were greater. Second, the study recruitment method captures only those participants who are still active some weeks into the course. MOOCs suffer from significant attrition rates, particularly in the first few weeks and it is not known why these learners dropped out. Third, this study provides no direct insight into any link between learning patterns and strategies reported and academic success. By working more closely with MOOC providers, it may be possible to gain access to participants at an earlier stage, and to gain the necessary ethical approval to link qualitative data to quantitative course data such as forum use, content access, and final mark. Combining self-report data with clickstream data can lead to more robust conclusions and has been used in online courses to study SRL (for example by Beheshitha, Gašević, & Hatala, 2015). Working more closely with providers must be managed sensitively however, as it is important that MOOC research is perceived as objective and independent of the MOOC provider. Fourth, this study utilised an interview script designed to elicit narrative descriptions of self-regulated learning that could be analysed with respect to the sub-processes of SRL described by Zimmerman (2000a), but for some sub-processes, the data collected was insufficient to allow extensive analysis. The different sub-processes of SRL are heavily interconnected and therefore it can be difficult examine these sub-processes in isolation. Meanwhile, some sub-processes, such as those relating to the reflection phase of SRL are inherently difficult to explore through interview without affecting the response of the participant. Interview questions could be further refined to make the instrument more effective. Alternatively, future studies could focus on individual sub-processes of SRL in detail. Further research could explore the efficacy of environments and tasks designed specifically to support the full range of learners who choose to study in MOOCs. By recognising and supporting the varied needs and skills of these learners, MOOCs can fulfil their potential to provide free, high quality learning for all.

## Figures and Tables

**Table 1:** Participant profile summary.

Participant ID	Gender	Location	Role
284	M	Nigeria	Data manager
334	F	USA	Paediatric pharmacist
152	F	Egypt	R&D innovation projects coordinator
358	M	Spain	Nurse teacher
366	F	USA	Lecturer
143	F	Spain	Epidemiologist
154	M	Qatar	ambulance nurse
340	F	Argentina	Research counsellor
394	M	Botswana	Medical laboratory scientist
280	F	Italy	Pharmacist
325	F	New Zealand	Data analyst
371	F	Spain	Psychiatrist
26	M	India	Clinical data curator
249	M	Peru	Neurologist
279	M	Uganda	Research nurse
72	F	Germany	Surgeon
24	M	Russia	Clinical research officer
256	F	United Kingdom	Pharmacist
318	M	India	Physician
275	F	USA	Clinical trials administrator
22	F	France	Medical epidemiologist
47	F	India	Clinical research associate
373	F	Serbia	Clinical research consultant
324	M	Brazil	Teacher
188	M	Thailand	Medical trainer
255	F	Belgium	Clinical trials project manager
28	M	Egypt	Physiotherapist
316	M	Saudi Arabia	Clinical pharmacy lecturer
226	F	India	Consultant (physician)
360	F	USA	Physician
295	F	India	Physician
287	M	India	Psychiatry lecturer
78	M	Romania	Physician
213	F	Taiwan	Physician
128	M	USA	Research coordinator
